# Regulating stem cell–based embryo model research in Japan: Proposals, debates, and future directions

**DOI:** 10.1038/s44319-025-00409-5

**Published:** 2025-03-14

**Authors:** Tsutomu Sawai, Shu Ishida, Chie Kobayashi, Yasuna Murase, Gyo Nakao, Tomonori Nakamura, Julian Savulescu

**Affiliations:** 1 Graduate School of Humanities and Social Sciences, Hiroshima University, Higashi-Hiroshima, Japan; 2 Uehiro Division for Applied Ethics, Graduate School of Humanities and Social Sciences, Hiroshima University, Higashi-Hiroshima, Japan; 3 Institute for the Advanced Study of Human Biology (ASHBi), Kyoto University, Kyoto, Japan; 4 Yong Loo Lin School of Medicine, National University of Singapore, Singapore, Singapore; 5 Graduate School of Arts and Sciences, The University of Tokyo, Tokyo, Japan; 6 Department of Anatomy and Cell Biology, Graduate School of Medicine, Kyoto University, Kyoto, Japan; 7 Hakubi Center for Advanced Research, Kyoto University, Kyoto, Japan; 8 Oxford Uehiro Centre for Practical Ethics, Faculty of Philosophy, University of Oxford, Oxford, UK; 9 Biomedical Ethics Research Group, Murdoch Children’s Research Institute, Melbourne, Australia; 10 Melbourne Law School, The University of Melbourne, Melbourne, Australia

**Keywords:** Economics, Law & Politics, Science Policy & Publishing, Stem Cells & Regenerative Medicine

## Abstract

Japan is developing regulations for stem cell–based embryo models, guided by domestic ethical considerations. Key challenges remain, including classification, oversight, and public engagement, highlighting the need for a balanced and transparent framework.

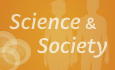

During the past years, research into embryo models—biological structures resembling embryos generated from pluripotent or somatic stem cells—has made remarkable progress. These so-called stem cell–based embryo models (SCBEMs) have the potential to advance our understanding of early developmental processes and the causes of various diseases, which may lead to new prevention measures and treatments. In 2017, the first mouse blastocyst model, known as “blastoid,” was successfully developed (Rivron et al, 2018), and, only 4 years later, human blastoid were generated from embryonic stem (ES) cells (Yu et al, 2021). This rapid scientific progress has sparked significant ethical debate about the moral status of SCBEMs and whether there is a need to oversee and regulate such research (Nuffield Council on Bioethics, 2024).

Several countries and international organizations have since moved to regulate research on human SCBEMs. Australia, for example, was one of the first jurisdictions that decided to treat blastocyst-like structures as equivalent to human embryos under the law (Australia NHMCR, 2021). In 2021, the International Society for Stem Cell Research (ISSCR) revised its guidelines for stem cell research and clinical applications, providing general criteria to guide each country in determining which types of human embryo model research should be subject to regulation (ISSCR, 2021; Box 1). The UK followed suit in 2024 by issuing rules for the generation and use of human SCBEMs, driven largely by researchers but also incorporating public feedback (Kinsella et al, 2024; Cambridge Reproduction, 2024; Box 2).

Asian countries that heavily invest in stem cell research already have regulations in place to permit and oversee research on SCBEMs (Box 3). Japan, too, has been exploring regulations at the national level, prompted by the rapid advances in SCBEM research and the revised ISSCR guidelines. This paper provides an overview of the recent discussions on regulation in Japan, collating its regulatory attempt with international trends, and identifies key challenges and future directions.

Japan, too, has been exploring regulations at the national level, prompted by the rapid advances in SCBEM research and the revised ISSCR guidelines.

## The emerging regulatory framework for SCBEM research in Japan

In 2023, the Japanese Cabinet Office on Bioethics established a working group “Creating Human-Embryo-Like Structures from Pluripotent Stem Cells.” Between August 2023 and March 2024, the group convened nine times and held two joint sessions with the Expert Panel on Bioethics, culminating in an interim report published in November (Expert Panel on Bioethics, 2024). According to this report, revisions to existing guidelines will take effect starting in the 2025 fiscal year.

Japan’s regulations concerning human embryos and stem cells are spread across multiple guidelines, which creates a complex web of rules (CSTI, 2004, see Table 1). One clear distinction is the different ethical requirements for ES cells versus other stem cells. For instance, research involving the *derivation* of human ES cells from embryos for regenerative medicine is regulated under the Guidelines on the Derivation of Human Embryonic Stem Cells, while the *storage* and *distribution* of human ES cells are covered by the Guidelines for the Distributing Institute of Human Embryonic Stem Cells. Researchers planning to *use* ES cells for research and/or clinical applications must follow the Guidelines on the Utilization of Human Embryonic Stem Cells. In contrast to the three guidelines, which jointly oversee human ES cell research at each stage, research involving human induced pluripotent stem (iPS) cells or somatic stem cells is generally guided by the Guidelines on the Research on Producing Germ Cells from Human iPS Cells or Human Tissue Stem Cells for generating germ cells, and otherwise by the Ethical Guidelines for Medical and Biological Research Involving Human Subjects.

**Table 1:** Overview of Human Embryo-Related Guidelines in Japan

Name	Year Established (Latest Revision)	Regulated Subject	Outline	Issuing Body	Remarks
Guidelines for Derivation of Human ES Cells	2019 (2022)	Human ES Cells	Ensure the scientific and ethical validity of the establishment and use of human ES cells.	MEXT & Ministry of Health, Labour and Welfare (MHLW)	Consolidates earlier guidelines into a unified framework as of 2014 and was updated further in 2019. Revision of prior guidance from 2001 and updated further in 2022.
Guidelines for the Distributing Institute of Human Embryonic Stem Cells	2019 (2022)	Human ES Cells	Rule that those involved in the distribution of human ES cells should make every effort to protect personal information.	MEXT	
Guidelines for Utilization of Human ES Cells	2019 (2022)	Human ES Cells	Prescribe the basic requirements to be observed from a bioethical point of view regarding the use of human ES cells.	MEXT	
Guidelines for conducting research to generate germ cells from human iPS cells or from human tissue stem cells	2010 (2023)	Human iPS Cells and Somatic Cells	Addresses ethical use and research concerning human iPS cells and other somatic cells.	MEXT	n/a
Ethical Guidelines for Medical and Biological Research Involving Human Subjects	2021 (2023)	Human iPS Cells and Somatic Cells	Covers ethical considerations for creating human germline cells.	MEXT, MHLW & Ministry of Economy, Trade and Industry (METI)	n/a
The Guidelines for Handling of a Specified Embryo	2001 (2024)	Human Embryos	Consolidated policy for research on human embryos.	MEXT	n/a
Basic Principles on the Handling of Human Embryos	2004	Human Embryos	Foundational document on ethical considerations and research scope for handling human embryos.	Cabinet Office	n/a
Guidelines for Research Using Gene-altering Technologies on Human Fertilized Embryos	2019 (2024)	Human Embryos	Ethical framework for reproductive and embryo modification technologies.	MEXT & MHLW	Includes updates reflecting advancements in genome editing technology.

Japan’s regulations concerning human embryos and stem cells are spread across multiple guidelines, which creates a complex web of rules.

Of these, the regulations most directly relevant to human SCBEMs—and hence facing imminent revisions—are the Guidelines on the Utilization of Human Embryonic Stem Cells and the Guidelines on the Research on Producing Germ Cells from Human iPS Cells or Human Tissue Stem Cells.

Box 1. The 2021 ISSCR guidelines In May 2021, the ISSCR released a revised edition of its *Guidelines for Stem Cell Research and Clinical Translation* (ISSCR, 2021). Developed over three years by a task force of scientists, ethicists and legal experts, the new guidelines provide a rigorous framework to address emerging ethical, scientific and policy challenges posed by recent breakthroughs in stem cell science—most notably, research on organoids, human–animal chimeric embryos and SCBEMs.

ISSCR guidelines classify research activities into three oversight categories: (1) those permissible under standard review mechanisms—for instance, most in vitro work with pluripotent stem cells; (2) those requiring specialized oversight owing to heightened ethical complexity, for instance in vitro culture of human embryos up to 14 days post-fertilization; and (3) those currently prohibited because of unresolved ethical and technical concerns such as heritable genome editing and human reproductive cloning. By distinguishing among these categories, the ISSCR underscores the necessity of aligning research practices with varying degrees of ethical scrutiny and societal values.

Notably, the generation of human SCBEMs that recapitulate the integrated development of a complete human embryo falls under category (2), thereby mandating a specialized oversight process. One of the most debated revisions in the 2021 Guidelines is the relaxation of the “14-day rule.” The updated policy now allows in vitro culture of human embryos beyond 14 days, provided there is compelling scientific justification and special oversight. This shift largely reflects technological advances and the growing feasibility of generating embryo-like entities without fertilization (Sawai et al, 2021).

Box 2. The 2024 UK code of practice for the generation and use of human stem cell–based embryo models In July 2024, the Cambridge Reproduction Research Centre, in collaboration with the Progress Education Trust, released the *Code of Practice for the Generation and Use of Human Stem Cell–Based Embryo Models* (Cambridge Reproduction, 2024). Guided by a multidisciplinary working group of scientists, ethicists and legal experts since March 2023, this initiative also incorporated public dialogue to capture diverse societal perspectives.

The Code addresses ethical considerations surrounding SCBEM research and applications, identifies regulatory gaps in the UK’s *Human Fertilisation and Embryology Act 1990*, and integrates public engagement to align scientific progress with social values. It provides detailed ethical guidelines, regulatory recommendations and strategies for sustained public involvement. Notably, by distinguishing human SCBEMs from conventional human embryos, the Code offers a flexible framework poised to accommodate ongoing scientific advances in this rapidly evolving field.

Although not legally binding, the Code serves as an authoritative reference for researchers, funders and institutions in the UK, and may also inform governance in international contexts. Its adoption seeks to promote the responsible and transparent conduct of SCBEM research, ensuring compatibility with existing regulatory principles.

Box 3. Regulatory frameworks for SCBEM research in East Asia Other research-intensive countries in East Asia, notably China and South Korea, also permit scientific research on human embryos. Beyond this general consensus, however, approaches to the governance of SCBEM differ markedly, reflecting each country’s historical trajectory and existing legislative frameworks.

China’s regulatory framework for human embryonic research is primarily guided by the Ethical Guidelines for Human Embryonic Stem Cell Research, issued jointly in 2003 by the Ministry of Science and Technology and the Ministry of Health (Peng et al, 2020). These guidelines require rigorous ethics committee oversight, informed consent protocols, prohibit reproductive cloning, and impose a 14-day limit for in vitro research—provisions broadly aligned with the ISSCR recommendations. In the absence of a national law dedicated to stem cell research, most oversight and enforcement takes place at the institutional level through internal review boards, although the degree of each varies across regions. In recent years, the government has indicated its intention to update regulations in response to rapid scientific developments, particularly in genome editing, underscored by the case of He Jiankui. Nonetheless, China’s legal system lacks explicit definitions for key embryo-related terms (Matthews and Moralí, 2020; Blasimme and Sugarman, 2023), potentially complicating the scope and applicability of future SCBEM regulations.

South Korea’s regulatory environment for human embryonic research is based on the Bioethics and Safety Act, enacted in 2005 and subsequently amended to address scientific progress and ethical concerns. This legislation arose partly in response to misconduct committed by stem-cell researcher Hwang Woo-Suk, prompting stricter oversight and transparency requirements.

The Act provides explicit legal definitions for “embryo” and related terms—which *may* also apply to SCBEMs (Blasimme and Sugarman, 2023)—and imposes relatively stringent controls on ES cell research (Song et al, 2024). For example, researchers must secure approval from both institutional review boards and national bioethics committees before conducting research involving human embryos or deriving ES cells; surplus in vitro fertilization embryos may be used for research until the appearance of a primitive streak rather than following a strict 14-day rule; somatic cell nuclear transfer is permitted solely for research; and reproductive cloning is categorically prohibited. The legislation also stipulates severe penalties for violations. Oversight is led by the Ministry of Health and Welfare, which periodically refines regulations to maintain alignment with scientific progress and international ethical standards. However, one notable limitation is the Act’s reliance on fertilization in its definition of “embryo,” thereby excluding SCBEM and similar embryoid research from its purview (Matthews and Moralí, 2020). Overall, South Korea’s framework is characterized by a relatively high degree of stringency, shaped by historical precedent and sustained efforts to uphold ethical integrity while fostering biomedical innovation.

## Key issues and proposed approaches

A key point during the debates of the working group was to determine how closely SCBEMs resemble actual human embryos. Traditionally, in Japan’s regulatory context, human embryos have been understood as “the buds of human life, potentially leading to the birth of a person if transferred into the uterus” (CSTI, 2004). Similarly, under the Act on Regulation of Human Cloning Techniques (Government of Japan, 2000), a “fertilized human embryo” is defined as “an embryo resulting from fertilization between a human sperm and a human oocyte.” In contrast, the working group views human SCBEMs as differentiated derivatives from human stem cells—excluding germ cells—that are therefore clearly distinct from natural developmental framework. Therefore, the regulatory frameworks for fertilized embryos do not apply to SCBEMs.

A related question raised by the working group was whether human SCBEMs should be included in the category “specified embryos”—a class of embryos designated under the Act of Regulation of Human Cloning Techniques and other guidelines—that requires stringent oversight to prevent the creation of human clones (MEXT 2024). Typically, “specified embryos” include human embryos derived by somatic cell nuclear transfer and human–animal hybrid embryos. The working group noted that, based on current scientific evidence, embryo models derived from ES or iPS cells—despite potentially sharing near-identical genetics with a donor—do not presently possess the capacity to develop into full-term offspring when transferred into a uterus. Therefore, they concluded, such models do not meet the definition of “specified embryos,” that is, cloned human embryos.

Accordingly, the working group posited that, at least in their current form, human SCBEMs differ substantially from both natural human embryos and cloned human embryos. However, the report states that if future advances enable SCBEMs to produce viable offspring—supported, for example, by robust evidence in mammals—additional regulatory measures may be warranted.

Given the present improbability of SCBEMs progressing to a fetal stage, the new Japanese regulations do not specify a maximum culture period, though periodic reviews will occur as ISSCR guidelines evolve. Instead, research institutions are expected to culture SCBEMs only as long as is scientifically necessary and to undergo ethics review on a case-by-case basis. Transferring SCBEMs into a uterus or any procedure likely to lead to birth is explicitly prohibited, and one major focus of institutional ethics committees will be to confirm that any project does not aim to produce a human individual.

Given the present improbability of SCBEMs progressing to a fetal stage, the new Japanese regulations do not specify a maximum culture period…

Another issue concerns informed consent. If researchers plan to generate new iPS cell lines from donor samples stored at their institutions, standard consent or an opt-out procedure is required. However, unlike research involving embryos created throug in vitro fertilization, no special consent protocol is mandated for the use of iPS or somatic stem cell–based embryo models. The adequacy of current consent guidelines for SCBEM research may warrant further discussion in light of donor autonomy, benefit-sharing concerns, and uses that may extend beyond the donor’s original scope of agreement.

## Ongoing challenges and future perspectives

A key strength of interim reports is their timeliness and alignment with international trends. Even though the reports do not have the legal force of a statute, they nonetheless shape national policy and establish a framework that is consistent with the ISSCR’s stance. Nonetheless, several critical issues remain unresolved.

Although scientists working on SCBEMs were invited to share their perspectives during the drafting process, opportunities for broader public participation were limited. Historically, Japan has relied on soliciting public comments prior to finalizing regulatory changes across various policy domains, effectively treating this mechanism as public engagement. However, empirical data on public awareness and attitudes toward SCBEM research remain scarce (Kiya et al, 2022). With the guidelines slated for implementation in the near future, there is still an opportunity to initiate more robust participatory efforts and incorporate a broader range of views.

By contrast, the UK code of practice for the generation and use of human SCBEMs was developed through an extensive public engagement process, which included public dialogues, surveys and community events aimed at gathering feedback from both stakeholders and the general public (Cambridge Reproduction, 2024). These efforts may offer valuable insights for designing and implementing more rigorous participatory approaches elsewhere. Furthermore, the introduction of AI-driven broad listening techniques could help capture an even wider range of perspectives in real time and facilitate more robust consensus-building.

… the UK code of practice for the generation and use of human SCBEMs was developed through an extensive public engagement process…

Because multiple guidelines apply depending on the type and intended use of stem cells (Table 1), there is a risk of confusion among researchers, ethics committees and regulators. Such a distributed system of regulations may also be an obstruction for researchers in Japan and institutions participating in international collaborative research. A more systematic approach that integrates regulations on both human embryos and SCBEMs may ultimately be necessary.

Because multiple guidelines apply depending on the type and intended use of stem cells […] there is a risk of confusion among researchers, ethics committees and regulators.

Furthermore, questions remain as to whether ethics committees in individual research institutions—responsible for reviewing a wide array of life-sciences research—are adequately prepared to address the nuanced ethical challenges posed by SCBEMs. In the USA, the need for specialized embryonic stem cell research oversight (ESCRO) committees has been repeatedly emphasized (Brewer and DeGrote, 2013; Chapman 2013; Devereaux and Kalichman, 2013; Ellison, 2013; Lomax, 2013), and a specialized ethics committee focused on SCBEMs has been proposed for the UK (Cambridge Reproduction, 2024). Although creating such committees in Japan may currently be not feasible due to limitations in human resources, there is a compelling case for careful deliberation on how best to provide oversight for research involving entities—SCBEMs—viewed by many as being “close to embryos.”

… questions remain as to whether ethics committees in individual research institutions […] are adequately prepared to address the nuanced ethical challenges posed by SCBEMs.

The USA has already seen legal disputes over informed consent related to SCBEM research (Mittleman, 2024). In Japan, the tentative conclusion that no additional consent measures are needed may face future scrutiny, especially concerning donor autonomy and the potential need for benefit-sharing if the research extends beyond the original scope of use. Although current guidelines do not require separate informed consent for SCBEM research, it is possible that future ethical and legal developments—both domestically and internationally—will force a re-evaluation of this position.

## Concluding remarks

This paper has provided an overview of the recent regulatory proposal surrounding SCBEM research in Japan. Under this new proposal, SCBEMs are treated as fundamentally distinct from fertilized embryos, and no universal limit on their culture duration is imposed. Instead, each project’s ethical suitability is evaluated by institutional ethics committees, in alignment with the ISSCR’s recommendations. Moreover, the proposed guidelines will be further informed and adapted in response to the global regulatory environment.

Despite these commendable developments, three critical issues persist: limited public engagement in the policymaking process, a patchwork of relevant regulations, and uncertainties surrounding informed consent. Addressing these challenges is imperative to ensure that Japan’s regulatory framework remains both ethically sound and adaptable to rapidly advancing scientific frontiers. By refining its guidelines, fostering public dialogue, and anticipating forthcoming breakthroughs, Japan could assume a leading role in promoting ethically responsible innovation in regenerative medicine and developmental biology.

Japan’s regulatory experience also provides broader lessons for other nations and for international stem cell governance. First, a unified regulatory framework that governs research materials across all stages—from their derivation and distribution to their eventual application—is preferable to separate guidelines for the generation, distribution and application of resources such as ES cells at discrete stages, which remains the case in Japan. Similarly, a coordinated approach encompassing diverse research materials and technologies, such as ES cells, iPS cells and cloning techniques, would foster a more cohesive regulatory environment, which is particularly important in emerging domains such as SCBEM research. A still more comprehensive framework might also encompass other entities, such as human organoids and non-human animals, given that parallel ethical questions—particularly those related to “moral status”—could arise in these contexts as well.

Regardless of whether such a comprehensive strategy is ultimately adopted, it will be essential to draw on the extensive ethical discourse surrounding human organoids and non-human animals to inform SCBEM research. In addition, in-depth discussions are needed to determine appropriate consent protocols at each stage of SCBEM research—ranging from cell collection to model generation, experimental use and eventual disposal. As in related realms of stem cell science, meaningful public engagement is vital to securing social consensus on the ethical use of SCBEMs and safeguarding the interests of cell donors, ultimately enhancing public acceptance and trust in SCBEM research.

## Supplementary information

Peer Review File
