# Supplementary material for: Regulating stem cell–based embryo model research in Japan: Proposals, debates, and future directions
Source: EMBO Rep. 2025 Mar 14;26(7):1682–7. doi: 10.1038/s44319-025-00409-5 (PMC11977183; doi:10.1038/s44319-025-00409-5)
Supplement: Supplementary file 1 — Peer Review File [file 44319_2025_409_MOESM1_ESM.pdf]

EMBO reports

Human Embryo Model Research in Japan: Ethics and Regulation

Tsutomu Sawai, Shu Ishida, Chie Kobayashi, Yasuna Murase, Gyo Nakao, Tomonori Nakamura, and Julian Savulescu

Corresponding author(s): Tsutomu Sawai (tstmsw@hiroshima-u.ac.jp)

Review Timeline:

Submission Date:	19th Feb 25
Accepted:	21st Feb 25

Editor: Holger Breithaupt

Transaction Report: This article was editorially reviewed.
